# A Unique Topoisomerase II Inhibitor with Dose-Affected Anticancer Mechanisms and Less Cardiotoxicity

**DOI:** 10.3390/cells10113138

**Published:** 2021-11-12

**Authors:** Zhi-Ying Li, Guang-Sen Xu, Xun Li

**Affiliations:** 1 Institute of Materia Medica, Shandong First Medical University & Shandong Academy of Medical Sciences, Jinan 250117, China; 2 Key Laboratory of Chemistry and Chemical Biology (Ministry of Education), School of Pharmaceutical Sciences, Cheeloo College of Medicine, Shandong University, Jinan 250012, China; 201936104@mail.sdu.edu.cn (Z.-Y.L.); shi.heng2009@163.com (G.-S.X.); 3 Key Laboratory of Forensic Toxicology, Ministry of Public Security, Beijing 100192, China

**Keywords:** acridone derivative 6h, topo II inhibitor, DNA intercalating agent, chemotherapy, less cardiotoxicity

## Abstract

Type II DNA topoisomerase (topo II) is an essential nuclear enzyme and a well-validated anticancer drug target. Previously, we have carried out several rounds of structural optimizations on our in-house topo II inhibitor **E17**, which was shown to have superior anticancer activity and less risk of multidrug resistance (MDR). Among the newly developed acridone derivatives, 6h displayed significant anticancer efficacy with unique mechanisms of action. At low concentrations, it arrested cancer cell cycles and triggered cell apoptosis, which is similar to the action of the well-known topo II inhibitor VP16. By contrast, 6h showed significant and long-term anti-proliferative activity at relatively high concentrations, with negligible influence on apoptosis. In addition, 6h exhibited no serious cardiotoxicity compared to doxorubicin (DOXO), a widely used topo II-targeting antineoplastic drug in clinic, but with damaging myocardial side effects. Collectively, our present work has supported the therapeutic value of 6h as a promising chemotherapy for cancers.

## 1. Introduction

Unwinding the duplex DNA induces differences in the topology and conformation of replicating DNA. DNA replication requires DNA breakage and religation to relieve the torsional stress, and this process is mediated by DNA nucleases topoisomerases (topos) [1]. Topos are usually divided into two major categories, topo I and topo II, which catalyze the passage of DNA single strands and double helices, respectively. Unlike topo I, ATP hydrolysis is necessary to maintain the biochemical functions of topo II by promoting topological transformations, rather than cleaving DNA recombination [2,3]. Albeit with distinct mechanisms, both topos function as prominent and well-acknowledged anticancer targets.

A plethora of topo II inhibitors, e.g., DOXO, etoposide (VP16), and mitoxantrone (MTX), have been developed as first-in-class chemotherapeutic drugs in clinic, in particular for the treatment of various advanced malignant tumors [4]. Nevertheless, these drugs continuously suffer limitations in cardiotoxicity, multidrug resistance (MDR), or therapy-induced secondary malignancies, all of which weaken their therapeutic values to a great extent [5]. Thus, there is an urgent need to develop more efficacious and safer topo II inhibitors with favorable druggable prospects.

Our lab has a long-standing interest and research record in the search for topo II-oriented antineoplastic agents, leading to the discovery of a spectrum of novel acridone, quinoxalinone, and anthraquinone derivatives with promising anti-proliferative efficacies [6,7,8,9]. Among them, the acridone derivative **E17** produced higher topo II inhibitory potency than two reference drugs, VP16 and ICRF-187. A preliminary mechanistic study unraveled that **E17** induced the accumulation of a cleavage complex without a significant influence on the degradation of topo II. The finding that **E17** might not cause serious MDR, unlike VP16, was more exciting, which inspired us to carry out further in-depth chemical optimizations on this lead compound (unpublished data). Thereafter, a more potent acridone-oriented topo II inhibitor 6h [1-((3-(dimethylamino)propyl)amino)-7-hydroxy-4-nitroacridin-9(10*H*)-one] was identified, which displayed stronger anticancer efficacy and unique dose-affected anticancer mechanisms compared to VP16.

Previously, several acridone derivatives have been identified as DNA intercalating topo II inhibitors [10,11], and their anticancer activity, topo II inhibition, and DNA intercalation effects are proven to be closely associated with cell cycle arrest and apoptosis. However, to the best of our knowledge, whether these two effects play the same role in affecting cell cycle arrest and apoptosis in acridone derivatives is still poorly understood. Although it is generally recognized that with increased concentrations of anticancer drugs exposed to tumor cells, more apoptosis will be induced and the cell cycle will be arrested at a particular phase, the question of how topo II inhibition and the DNA intercalation of DNA intercalating topo II inhibitors affect anticancer activity still needs to be explored in depth.

To this end, we herein present the biological evaluation and mechanistic study results of 6h, which were quite different from those of VP16 and existing DNA intercalating topo II inhibitors.

## 2. Materials and Methods

### 2.1. Materials

**Figure 1:**
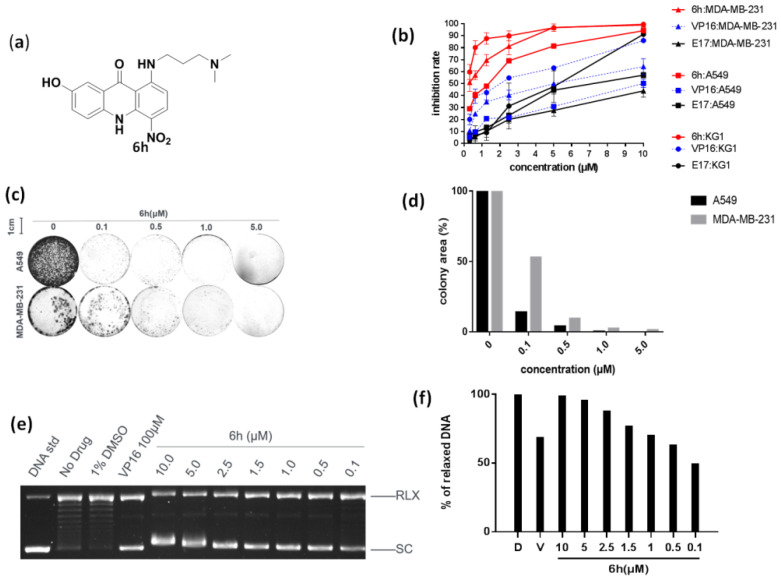
6h exhibited strong anticancer efficacy by inhibiting topo IIα. (**a**) Chemical structure of 6h. (**b**) 6h dose-dependently inhibited the proliferation of different cancer cells. Data are presented as mean ± SD (*n* = 3). (**c**,**d**) 6h inhibited colony formation of two cancer cells. Images display the colony formation in 6h-treated cells. 6h dose-dependently inhibited colony formation in A549 and MDA-MB-231 cells. (**e**,**f**) 6h inhibited topo IIα-mediated DNA relaxation. VP16 was set as a positive control. Supercoiled pBR322 DNA (SC) and relaxed DNA (RLX) are shown. D: DMSO, V: VP16 at 100 μM.

The chemical structure of 6h is presented in Figure 1a. Reference drugs (VP16 and DOXO) were commercially available from Sigma-Aldrich Corporation, and another reference compound **E17** was available from our in-house chemical library.

### 2.2. Cell Cultures

All cells were commercially obtained from the Chinese Academy of Sciences Cell Bank. Human breast cancer cells, MDA-MB-231, were maintained in L-15 medium, while human lung cancer cells (A549), human acute myelogenous leukemia cells (KG1), and rat myocardial cells (H9c2) were maintained in RPMI 1640 medium. Both media were supplemented with 10% fetal bovine serum (FBS), 100 U/mL penicillin, and 100 mg/mL streptomycin. Cells were cultured at 37 °C in a humidified atmosphere containing 5% CO_2_.

### 2.3. Cell Viability Assay

Cell proliferation was measured using the Cell Counting Kit-8 (CCK-8) method, based on the manufacturer’s instructions. Briefly, cells were incubated in a 96-well plate at a density of 3~15 × 10^3^ cells/well/100 μL for 12 h in 5% CO_2_ incubator at 37 °C. Then, cells were treated with different concentrations of compounds for another 72 h. Finally, 10% CCK-8 was added to each well and incubated for an additional 4 h. Absorbance was measured at 450 nm. The inhibitory rate was calculated and the cytotoxicity of target compounds was expressed as IC_50_ using GraphPad Prism 7.

### 2.4. Colony Formation Assay

Cells were seeded in a 6-well plate at a 1 × 10^4^ cell/well density and treated with increasing concentrations of compound 6h for 24 h. Then, fresh medium was replaced, and cells were incubated for additional 7 days. Thereafter, media was discarded, 1 mL of MeOH:AcOH (3:1, *v*/*v*) was added, and the mixture was fixed at −20 °C for 1 h. After the solution was discarded, plates were air-dried and the cells were stained with 2 mL 1 × Giemsa stain solution at room temperature for 30 min. The plates were imaged using a gel imaging instrument (ZEISS, Jena, Germany). Image-Pro Plus was used to calculate the colony formation area, and the untreated group of each cell was set to 100% in colony formation. The result was analyzed to generate histogram using software Prism 7 [12,13].

### 2.5. Topo IIα-Mediated pBR322 DNA Relaxation Assay

Supercoiled pBR322 (0.25 μg) was incubated with 1U IIα for 30 min at 37 °C in relaxation buffer (5 mM Tri-HCl, 12.5 mM NaCl, 1 mM MgCl_2_, 0.5 mM DTT, 10 μg/mL albumin, 1 mM ATP, pH 7.5) in the presence of increasing concentrations of 6h or VP16. The reaction was terminated by adding 2 μL of 10% SDS, and then the samples were electrophoresed through a 1% (*W*/*V*) agarose gel at 100V for 1 h. The gel was stained with ethidium bromide (1 μg/mL in water) for 15 min, destained in water for 10 min, and visualized with a gel documentation system (Bio-Rad ChemiDoc™ XRS⁺, Hercules, CA, USA).

### 2.6. Measurement of Mitochondrial Membrane Potential (∆ψ_m_)

The H9c2 cells were seeded on 6-well plates overnight. Cells were treated with 6h or DOXO for 24 h. Then, 10 μg/mL of JC-1 dye was added to the media and incubated for another 20 min at 37 °C. After removing the dye, cells were washed with PBS buffer twice and immediately examined under a fluorescence microscope.

### 2.7. Cell Cycle Analysis

Cells were treated with 6h or VP16 for 24 h, harvested in cold PBS buffer, fixed in 70% ethanol, stored overnight at 4 °C, washed with PBS again, resuspended in 1 mL of 20 mg/mL PI staining reagent containing 100 mg/mL RNase, and incubated in dark for 30 min. Cell cycle distributions were assessed by flow cytometry (Agilent NovoCyte, Santa Clara, CA, USA).

### 2.8. Annexin V-FITC/PI Apoptosis Detection Assay

KG1 cells were treated with 6h or VP16 for 48 h, harvested, washed with PBS twice, resuspended in 100 μL of incubation buffer containing Annexin V-FITC and PI, incubated in dark for 20 min, and analyzed by flow cytometry (Agilent NovoCyte, USA).

### 2.9. Data Analysis

The inhibiting rate of 6h or VP16 to topo IIα was calculated with software ImageJ and the colony formation area was counted with software Image-Pro Plus 6.0. Data that came from flow cytometry were analyzed with NonoExpress. The experiments were repeated at least three times. Quantitative data are presented as means ± standard error of mean (SEM). A *p* value < 0.05 was considered significant.

## 3. Results

### 3.1. 6h Exhibited Significant Anti-Proliferative Activity by Inhibiting Topo IIα

The anti-proliferative activity of 6h was initially examined towards three types of topo II-sensitive cancer cells (MDA-MB-231, A549, and KG1), and VP16 and **E17** were utilized as positive controls. As demonstrated in Figure 1b, 6h displayed stronger anticancer efficacy than VP16. When treated with 5.0 μM of 6h, the growth inhibition was around 80% in A549 cells. By contrast, the inhibition rate of VP16 was lower than 30% under the same condition. The same phenomenon was observed in different concentrations and for different cancer cells. It is worth noting that 6h demonstrated approximately 10 times more potent cytotoxicity against the test cancer cells than VP16.

The cytotoxic effect of 6h was further confirmed by a colony formation assay (CFA), which is a well-acknowledged in vitro cell survival assay based on the colony formation ability of a single cell [14]. Two adherent cells, MDA-MB-231 and A549, were used in this assay. As delineated in Figure 1c,d, the colony formation area was less than 10%, even in the presence of 6h at 0.5 μM. Additionally, A549 cells seemed to be more sensitive at a low dosage of 6h compared to MDA-MB-231 cells.

Given the fact that the relaxation of a supercoiled DNA substrate is dependent on topo II, a dose-dependent DNA relaxation assay, in the presence of topo IIα and 6h, was performed by using the supercoiled plasmid pBR322 as a substrate. As evinced in Figure 1e, the majority of pBR322 existed in a supercoiled state, proving that 6h strongly prevented topo IIα-mediated DNA relaxation. The inhibition rate of 6h approached 70.78% at 0.5 μM and 49.36% at 0.1 μM, while that of VP16 was 68.56% at 100 μM. This result indicated that 6h behaved as a more potent anticancer agent than VP16.

### 3.2. 6h Induced Different Cell Cycle Arrests in Cancer Cells

**Figure 2:**
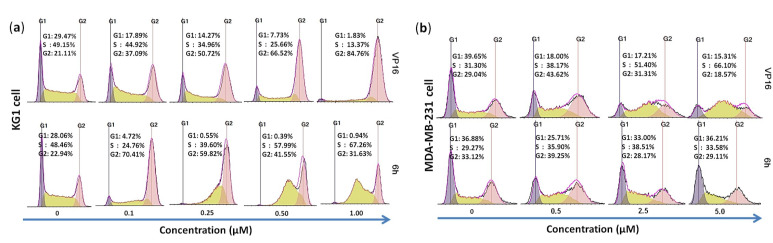
(**a**) Topo II inhibitors 6h and VP16 induced G2/M phase arrest in KG1 cells. Cancer cells were treated with different concentrations of 6h (upper panel) or VP16 (lower panel) for 24 h and subjected to cell cycle analysis. (**b**) VP16 and low concentration of 6h induced S phase arrest in MDA-MB-231 cells while high concentration of 6h gave a different result.

Next, we hoped to examine how 6h affected the cell cycle progression of KG1 cells by a flow cytometry assay. As shown in Figure 2a, the number of KG1 cells in the G2/M phase dramatically increased from 22.94% to 70.41% in the presence of 6h at 0.1 μM, which was equivalent to VP16 (21.11% to 84.76% at 1.0 μM). These results further verified that 6h could induce serious cell cycle arrest, and its activity was comparable to VP16.

To double check the cell cycle arrest, 6h and VP16 were subsequently treated in MDA-MB-231 cells under the same conditions. It was found that the cell cycle of VP16 was arrested in the S phase, and the cell number increased from 31.30% to 66.10%. As far as 6h was concerned, unexpectedly, the cell number in the S phase only slightly increased by 10% at 2.5 μM, and almost no change was observed at a high concentration (5.0 μM), as evinced in Figure 2b. It is likely that the cell cycle of 6h-treated cells was blocked at the initial state in MDA-MB-231 cells, which differed entirely from the cell cycle of VP16-treated cells.

### 3.3. 6h Induced Obvious Apoptosis in KG1 Cells

**Figure 3:**
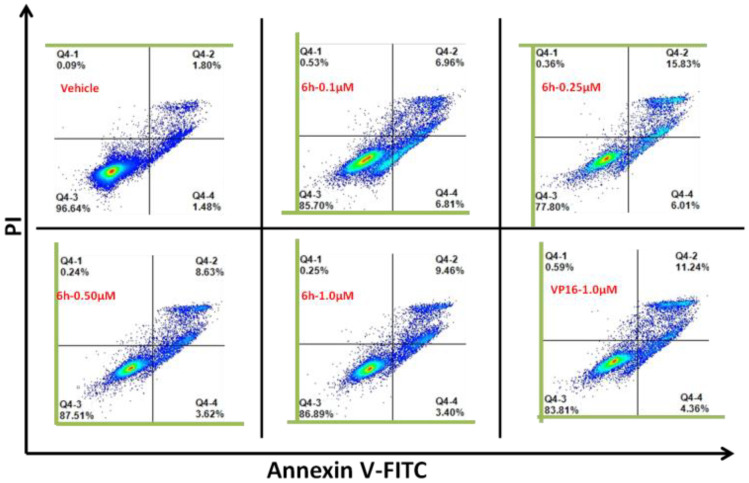
6h could induce significant apoptosis in KG1 cells. After exposure to 6h for 48 h, cells were harvested and stained with Annexin V-FITC and PI, and then analyzed by flow cytometry to determine early or late apoptotic and necrotic cell populations.

To check whether the observed prominent cytotoxicity of 6h was associated with apoptosis, an Annexin V/PI detection assay was performed in KG1 cells. As illustrated in Figure 3, when KG1 cells were exposed to 0.25 μM of 6h, the proportion of apoptotic cells increased from 3.28% to 21.84%, which was still more than VP16 (15.6%) at a relatively higher concentration of 1.0 μM. The results demonstrated that 6h could induce significant cell apoptosis. However, with increasing concentrations of 6h, the number of apoptotic cells decreased, which might be largely attributed to the DNA intercalation nature of 6h, and this still needs further verification.

### 3.4. 6h Induced Less Cardiotoxicity in H9c2 Cells

Anthracyclines, such as DOXO, epirubicin, and idarubicin, still remain mainstays as first-line chemotherapeutic drugs, with tangible effectiveness spanning from hematological malignancies to varied solid tumors. However, anthracycline-related cardiotoxicity, in a dose-dependent manner, has drastically restricted their wide clinical applications [15,16]. Cardiac toxicity detection of 6h was, therefore, conducted by examining H9c2 cardiac myoblast cells from rats.

**Figure 4:**
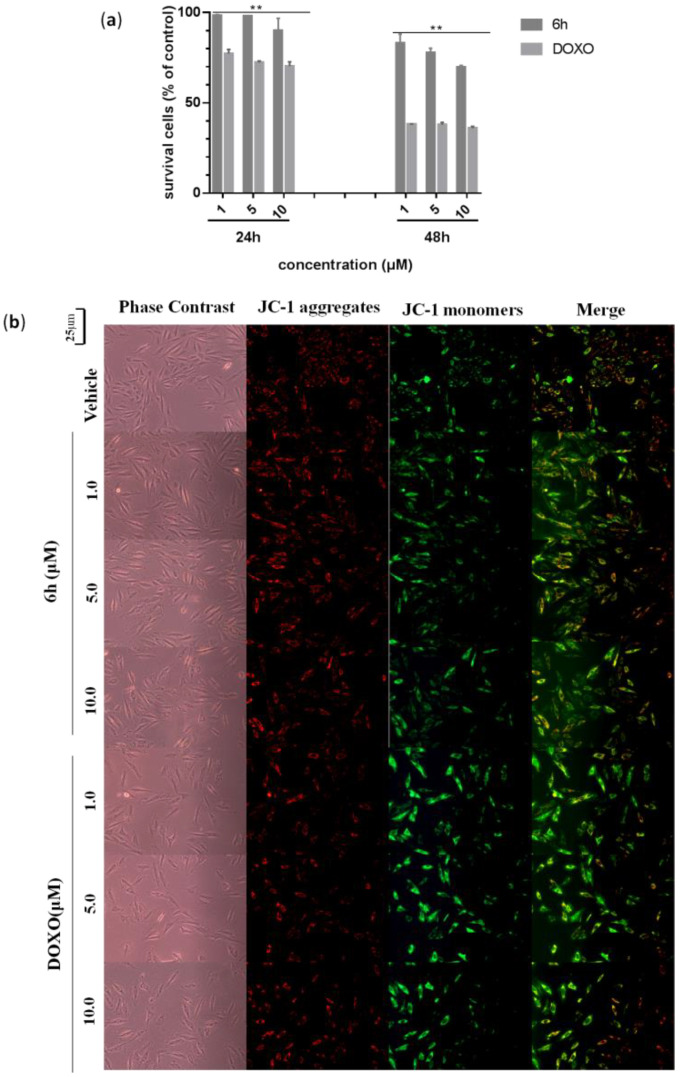
6h induced less cardiotoxicity in H9c2 cells. (**a**) 6h induced less cardiac cell cytotoxicity. H9c2 cells were treated with 6h or DOXO for 24 h or 48 h, and cell viability was detected by CCK-8 method. Means ± SEM; *n* = 3; **: *p* < 0.01, 6h vs. DOXO. (**b**) 6h did not induce mitochondrial damage in H9c2 cells. H9c2 cells were maintained in media with or without 6h or DOXO for 24 h. Cells were stained with JC-1 and examined under a fluorescence microscope.

As shown in Figure 4a, cell vitality was comparable to the control group in the presence of 6h at 1.0 μM and 5.0 μM for 24 h. When the concentration of 6h was increased to 10 μM, the cell survival rate was approximately 90%, while the DOXO-treated group exhibited a cell survival rate of 70% at the same concentration. After treatment of 6h for 48 h, the cell survival rate dropped to 80% at 1.0 μM, and 70% at 5.0 μM or 10.0 μM; therefore, it seems very unlikely that 6h will damage cardiac cells. However, obvious cell damage was observed in the DOXO group after 48 h of exposure, where the cell survival rate dropped to 36~38%.

Many studies have suggested that mitochondrial dysfunction is not only the primary molecular mechanism underlying DOXO-induced cardiotoxicity, but also largely contributes to the intrinsic and extrinsic apoptosis [17,18,19]. Therefore, we examined the influence of 6h on mitochondrial membrane potential (∆*ψ*_m_) using a JC-1 staining assay. As shown in Figure 4b, after the treatment of H9c2 cells with different concentrations (1, 5 and 10 μM) of 6h, red granular aggregates were completely observed in the cytoplasm, implying that 6h did not cause any changes in ∆*ψ*_m_. However, significant loss of ∆*ψ*_m_ was observed in the DOXO-treated group. In addition, the cytoplasm was wrinkled, distorted, and broken in the control group. In contrast, the cytoplasm of 6h-treated H9c2 cardiac cells was still plump and displayed less damage, which is indicative of the lower cardiotoxicity of 6h.

## 4. Discussion

Topo II inhibitors still remain first-in-class chemotherapeutic regimens in clinic, with well-recognized effectiveness for the treatment of both solid tumors and hematopoietic malignancies [20,21]. However, long-term therapy-induced side effects, cardiotoxicity in particular, have greatly hampered their clinical applications. The DNA intercalator 6h is a recently identified acridone derivative in our group; it comes from several rounds of structural optimizations of E17, a previously well-characterized topo II-targeting lead compound with potent anti-proliferative activity and less possibility of triggering MDR. The biological evaluation and mechanistic study of 6h revealed that it exhibited more potent anti-proliferative efficacy and stronger topo IIα inhibition compared with VP16 and **E17**.

Its unique dose-affected anticancer mechanisms are more intriguing. At low concentrations, 6h could arrest the cell cycle and induce obvious apoptosis in MDA-MB-231 cells, which is very similar behavior to that of VP16. However, at a relatively higher concentration, the cell cycle stayed at the initial state and the apoptosis decreased in KG1 cells, which might be largely attributable to its strong DNA intercalating ability (unpublished data). By contrast, a high concentration of 6h is supposed to limit the function of topo II and the accumulation of DNA double-strand breaks (DSBs); this is based on the fact that DNA intercalators can insert into the base pairs of DNA and prevent topo II from binding to the DNA, while the accumulation of DSBs is the direct cause of topo II inhibitor-induced apoptosis, as has been described by Tewey et al. [22,23]. In a word, unlike VP16, with high concentrations, the DNA intercalator 6h may impede topo II from interacting with DNA, leading to different cell cycle arrest and apoptosis, accordingly.

Meanwhile, the reduced apoptosis of KG1 cells, by treating them with high concentrations of 6h, might be largely attributable to the observed fact that at the time when 6h, a strong DNA intercalator, triggered cell apoptosis, it was posterior to the non-intercalator after 48 h of treatment. To the best of our knowledge, this is the first report of the different parts that topo II inhibition and the DNA intercalation of an acridone-based DNA intercalating topo II inhibitor play in the anticancer activity of 6h, which might also be applied to other strong DNA intercalators. This systematic and mechanistic study is still being investigated in our lab, and we will report its progress in the near future.

Rat H9c2 cardiomyocytes were used to evaluate the cardiotoxicity of 6h. As 6h did not induce mitochondrial injury, and has little effect on cell viability, this indicates that 6h would not cause serious cardiotoxicity, unlike DOXO.

## 5. Conclusions

Our newly developed acridone derivative 6h is a strong topo II inhibitor. It arrests cancer cell cycles and induces cancer cell apoptosis at low concentrations, but strongly inhibits cancer cell proliferation at high concentrations, which suggests that 6h has long-term, effective anticancer activity, and unique dose-affected anticancer mechanisms. Furthermore, 6h does not damage the mitochondria of cardiac cells, such as anthracyclines, further supporting the low cardiotoxic effect of 6h. Altogether, 6h is a promising topo II-mediated anticancer agent and worthy of further development.
